# Farmers’ livelihood strategies and perceived constraints from poor and non-poor households: A dataset from a field survey in Nghe An, Vietnam

**DOI:** 10.1016/j.dib.2021.106991

**Published:** 2021-03-31

**Authors:** Quan-Hoang Vuong, Phu Pham, My-Hien Nguyen, Cong-Thang Ngo, Phuong-Mai Tran, Quy Van Khuc

**Affiliations:** a Centre for Interdisciplinary Social Research, Phenikaa University, Hanoi 100000, Vietnam; b Institute of Ecology and Biological Resources, Vietnam Academy of Science and Technology, 18 Hoang Quoc Viet, Cau Giay, Hanoi, Vietnam; c Vietkaplab, Vietkap group, Hanoi 100000, Vietnam; d Department of Economics, National Economic University, Hanoi 100000, Vietnam; e Department of International Business, Foreign Trade University, Hanoi 100000, Vietnam; f Faculty of Economics and Business, Phenikaa University, Hanoi 12116, Vietnam

**Keywords:** Rural livelihood, Plantation forests, Primary data, Sustainable rural development, Vietnam

## Abstract

The first Sustainable Development Goals (SDGs) of The United Nations aims to “end poverty in all its forms everywhere”. Its seven associated targets aim, among others, to eradicate extreme poverty for all people everywhere. In Vietnam, poverty eradication in ethnic minorities and mountainous areas are among the top priorities. This study aims to learn about farmers’ livelihoods associated with perceived difficulties in Chau Thai Commune, Nghe An Province, a rural mountainous area in Vietnam. A random sampling technique and a face-to-face interview method were employed to conduct a field survey in the region in 2018. The dataset collected from 215 households shows that Chau Thai Commune's livelihood largely depends on agriculture and forestry. Plantation forest and livestock are major sources of farmers’ income while forestland accounts for over 90% of households’ land. Besides, the disparity in livelihood in areas such as forestland, labor and income between the poor and non-poor households is reported. This primary data could be useful for scholars who want to conduct a further in-depth study and or experts, policymakers who work in Vietnam's ‘New Rural Development’ program to devise a better rural livelihood -improvement policy for farmers, particularly the poor in the uplands of Vietnam and beyond.

## Specifications Table

**Table:** 

Subject	Economics, Econometrics and Finance, Environmental Science
Specific subject area	Economic Development and Growth, Management, Monitoring, Policy and Law
Type of data	Table Figures Excel files
How data were acquired	Data were collected using a field survey. A questionnaire-based face-to-face interview method was employed to survey households during February 2018. Data were converted to .xlsx format for formal analysis in SPSS version 22
Data format	Raw Analyzed
Parameters for data collection	The survey's target respondents were residents living in Chau Thai commune, Nghe An Province, Vietnam, including 4 villages Dong Minh, Ban Hat, Thai Quang, and Dong Hin.
Description of data collection	The study was conducted through a field survey in Nghe An using a random sampling technique
Data source location	Information was obtained from Nghe An (longitude 105.077833, latitude 19.298972), Vietnam.
Data accessibility	Repository name: Mendeley repository • Data identification number: DOI: 10.17632/7bf279kgfg.1 • Direct URL to data: http://dx.doi.org/10.17632/7bf279kgfg.1

## Value of the Data

• The primary dataset will be useful for researchers who want to learn about rural livelihood and its influencing factors between  poor and non-poor households in Chau Thai, Nghe An.
• The primary dataset will be helpful for researchers who wish to conduct comparative studies on the distribution of plantation forests in Chau Thai, Nghe An Province, with different provinces or different countries worldwide.
• The constructed dataset will help agricultural economists and/or policymakers who seek science-based solutions and/or design more appropriate policies for new rural development and poverty amelioration.

## 1 Data Description

Forests play a vital role in people's livelihoods, especially for poor people living in remote and upland areas. Accordingly, forest development has been a high priority in many parts of the world including Vietnam [1], [2]. Despite tremendous efforts by the government, further rural development is currently hindered by the existing livelihood and forestry policies, stemming from a limited understanding and/or the lack of scientific advice [1], [3], [4]. As the largest province in the country, Nghe An not only experiences a high rate of deforestation and forest degradation [5], but also has a great deal of potential for afforestation and reforestation, which has attracted substantial investment in climate change mitigation projects, contributing to emission reduction. In addition, living in a poor, mountainous commune, the indigenous people's income largely depends on agricultural production and forest and non-timber forest products (NTFPs) [6], [7]. Therefore, a better understanding of rural livelihood associated with forest planting-related constraints is crucial to devise better sustainable rural development and climate change mitigation policies. For these reasons, Chau Thai was chosen as the study region. In 2018, households living within 4 villages were surveyed through a questionnaire consisting of 62 items.

After eliminating some incomplete answers, our data presented in this study includes 215 observations with information on three aspects: (1) resource structure and the local people's livelihood strategies, (2) factors hindering production forest planting, (3) the difference between poor and non-poor households, and (4) the personal information of the head of households. It is noted that households are categorized into poor and non-poor according to Vietnam's poverty line in rural areas between 2016 and 2020 ($1.02 per person/day) [8], [9]. As sustainable rural development and climate change mitigation programs associated with afforestation and reforestation remain the nation's top priority, the findings associated with the data of our research aims to facilitate policymakers and governments to devise a better sustainable economic development policy in Vietnam and other places in the world. Following are brief results of the research.

**Fig. 1:**
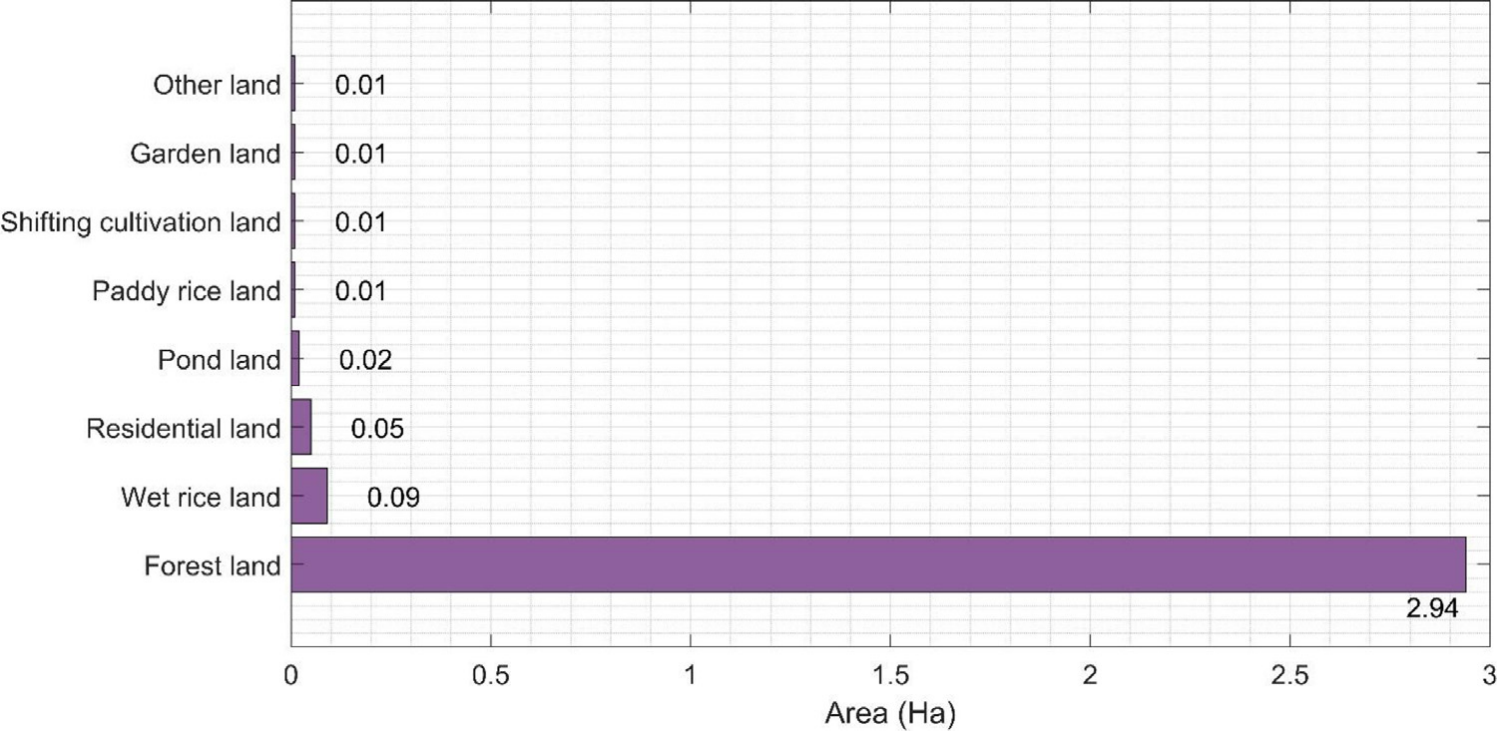
Types of land use of surveyed households in Chau Thai Commune.

Table 1 presents the distribution of economic factors amongst households in Chau Thai, Nghe An. Most of the households’ land area is forestland, accounting for over 90% (Fig. 1). Revenue from plantation forests and livestock farming were dominant income sources for the households. Specifically, on average, the revenue of livestock and plantation forests were $769.27 and $654.59, respectively, ranking second and third, whereas wages were recorded to the dominant source of household income ($1184.69). However, the status of forestland ownership varied widely for the difference of land possession was 12.34 hectares. In addition, the loan policies in Chau Thai exerted a considerable influence on the locals, with roughly 50% of whom took out loans. The number of people in a family also played a significant part in livelihood strategies. The average number of members was 4, while the average labor force was 2.45, that is, on average, one person would have to feed another. Besides, a relatively gender-balanced workforce was recorded. However, the income inequality was marked when the disparity between the richest and poorest households was $9112.04 per year.

**Table 1:** Land use, income structure, and livelihood strategies.

							95% Confidence Interval for Mean			
Farmers’ livelihood strategies		N	Mean	Std. Deviation	Std. Error	Min	Lower Bound	Upper Bound	Max	Range
Land use	Total area (ha)	215	3.15	2.86	0.20	0.11	2.77	3.54	12.45	12.34
	Wet rice land area (ha)	215	0.09	0.06	0.00	0.00	0.09	0.10	0.44	0.44
	Paddy rice land area (ha)	215	0.01	0.15	0.01	0.00	−0.01	0.03	2.00	2.00
	Shifting cultivation land area (ha)	215	0.01	0.10	0.01	0.00	0.00	0.02	1.00	1.00
	Garden land area (ha)	215	0.01	0.03	0.00	0.00	0.01	0.02	0.23	0.23
	Forestland area (ha)	215	2.94	2.80	0.19	0.03	2.57	3.32	12.00	11.97
	Pond land area (ha)	215	0.02	0.07	0.01	0.00	0.01	0.03	1.00	1.00
	Residential land area (ha)	215	0.05	0.08	0.01	0.00	0.04	0.06	1.00	1.00
	Other land area (ha)	215	0.01	0.14	0.01	0.00	−0.01	0.03	2.00	2.00
Loan	Taking out a loan from banks for production	215	0.50	0.50	0.03	0.00	0.43	0.57	1.00	1.00
	Borrowing money from friends for production	215	0.35	0.48	0.03	0.00	0.28	0.41	1.00	1.00
Labor	Number of family members	215	4.68	1.55	0.11	1.00	4.47	4.89	11.00	10.00
	Number of males	215	2.39	1.06	0.07	0.00	2.24	2.53	7.00	7.00
	Number of females	215	2.27	1.05	0.07	0.00	2.13	2.42	7.00	7.00
	Number of working people	215	2.45	1.05	0.07	0.00	2.31	2.59	6.00	6.00
Social relationship	Holding position in a local organization	215	0.12	0.32	0.02	0.00	0.07	0.16	1.00	1.00
Revenue	Total income ($)	215	3034.22	1784.39	121.82	385.08	2794.61	3274.28	9497.12	9112.04
	From rice ($)	215	224.44	149.93	10.26	0.00	204.36	244.52	963.81	963.81
	From corn ($)	215	1.78	17.40	0.89	0.00	0.00	3.57	160.64	160.64
	From potato ($)	215	0.89	8.48	0.45	0.00	−0.45	2.23	107.09	107.09
	From cassava ($)	215	1.34	12.49	0.89	0.00	−0.45	2.68	178.48	178.48
	From soybean ($)	215	2.23	21.86	1.34	0.00	−0.89	4.91	267.73	267.73
	From fruit ($)	215	43.73	268.17	18.29	0.00	7.59	79.87	3257.33	3257.33
	From livestock ($)	215	769.27	783.10	53.55	0.00	663.96	874.57	5019.86	5019.86
	From seafood ($)	215	19.63	66.04	4.46	0.00	10.71	28.56	468.52	568.52
	From forest ($)	215	654.59	677.35	46.41	0.00	563.56	745.62	3718.26	3718.26
	From NTFPs ($)	215	1.34	18.29	1.34	0.00	−1.34	3.57	267.73	267.73
	From wages (labor) ($)	215	1184.69	1331.49	91.03	0.00	1005.31	1363.62	6425.42	6425.42
	From doing business ($)	215	37.93	266.83	18.29	0.00	2.23	73.62	3212.71	3212.71
	From official salary ($)	215	45.07	314.13	22.31	0.00	0.89	89.69	4283.61	4283.61
	From pension ($)	215	48.19	315.02	21.42	0.00	5.80	90.58	3480.43	3480.43

NTFPs: non-timber forest products.

Table 2 provides a comparison of residents’ assessments of the limitations in planting forest in Chau Thai commune. The table delineates four groups of categories, including land, loan, labor, and income [10]. Although forests play an important role in households’ livelihoods, exploring and harnessing forestland for the good of their family as a whole are major challenges, rated 3.25/5, due to a small area, degraded land, steep slopes, arable land far from farm home and land without Land Ownership Certificate.

**Table 2:** Farmers’ perceived constraints about household livelihood.

							95% Confidence Interval for Mean			
Farmers’ perceived constraints		N	Mean	Std. Deviation	Std. Error	Min	Lower Bound	Upper Bound	Max	Range
Land	Difficulty with forestland	215	3.25	1.27	0.09	1.0	3.08	3.42	5	4
	Small forestland	215	4.20	1.07	0.07	1.0	4.05	4.34	5	4
	Poor and infertile forestland	215	2.76	1.34	0.09	1.0	2.58	2.94	5	4
	Faraway location	215	2.69	1.45	0.10	1.0	2.49	2.88	5	4
	Steep	215	3.94	1.08	0.07	1.0	3.80	4.09	5	4
	Inability cultivate	215	2.67	1.58	0.11	1.0	2.46	2.89	5	4
Loan	Difficult to access loans (from banks, friends,etc.)	110	2.35	1.26	0.12	1.0	2.11	2.58	5	4
	Little loan availability	110	3.00	1.76	1.28	1.0	2.76	3.24	5	4
	Short loan period	110	3.35	1.06	0.10	1.0	3.14	3.55	5	4
	High interest rate	109	3.26	1.09	0.10	1.0	3.05	3.46	5	4
	Little or no capital	164	3.90	1.08	0.08	1.0	3.74	4.07	5	4
Labor	Difficulty with human resources	215	3.70	1.122	0.08	1.0	3.55	3.85	5	4
	Small number of workers in the family	215	3.86	1.09	0.07	1.0	3.71	4.01	5	4
	Poor health	215	3.39	1.24	0.08	1.0	3.22	3.56	5	4
	Poor education	215	3.67	1.20	0.08	1.0	3.51	3.84	5	4
	Little experience and knowledge	215	3.60	1.17	0.08	1.0	3.45	3.76	5	4
	A fragile relationship with people around	215	3.27	1.27	0.09	1.0	3.10	3.45	5	4
Revenue	Difficult to sell forest products	215	2.64	1.397	0.10	1.0	2.45	2.83	5	4
	Unclear prices of forest products	215	3.53	1.37	0.09	1.0	3.35	3.72	5	4
	Unstable and volatile prices of forest products	215	3.96	1.00	0.07	1.0	3.82	4.09	5	4
	Low prices of forest products	215	3.70	1.15	0.08	1.0	3.55	3.86	5	4
	Hard bargains from wholesalers	215	2.73	1.48	0.10	1.0	2.53	2.93	5	4
	Weak manufacturing market	51	2.84	1.16	0.16	1.0	2.52	3.17	5	4

NTFPs: non-timber forest products.

Moreover, people in Chau Thai commune faced many difficulties in taking out loans. Despite easy access to loans, the mean values from 3.00 to 3.35/5 on grounds of short loan repayment period, high interest rate and limited amount of money lent. More importantly, most families had no or little capital. Labor is also an essential factor in the quality of economic development of the region. In Chau Thai, generally, the labor force had many limitations in both quantity and quality. On top of production factors, the market for forest products also posed major obstacles for indigenous households. For example, the statement that prices of forest products were unstable and volatile with the reported average score is 3.96/5.

Table 3 compares and contrasts two groups of households with income levels in Chau Thai, Nghe An. Overall, these groups bore little resemblance to each other when taking land area, loan getting, family workforce, and income sources into consideration. First, the income gap between poor and non-poor households was relatively high, at approximately $2000 per year. This disparity mainly came from plantations, labor, livestock farming, and crop cultivation. The most significant difference was in wage earnings, at $1171.75, generating $839.32 greater than the second-main income source - planted forests. The information on the role of forests could be partly explained by the fact that the area of forest owned by each group was different. According to the statistics presented here, the average land area of non-poor households was much larger than that of their poor counterpart regarding all types of land surveyed. A gap of 1.51 ha in forestland made the most significant contribution to this difference.

**Table 3:** Differences in livelihood capitals between poor and non-poor households.

		Levene's Test for Equality of Variances		T-test for Equality of Means						
						Sig.	Mean	Std. Error	95% Confidence Interval of the Difference	
Farmers’ livelihood strategies		F	Sig.	t	df	(2-tailed)	difference	Difference	Lower	Upper
Land use	Total area (ha)	18.46	0.00	3.99	213.00	0.00	1.59	0.35	0.90	2.28
	Wet rice land area (ha)	0.01	0.93	0.37	213.00	0.71	0.00	0.01	−0.01	0.02
	Paddy rice land area (ha)	3.54	0.06	0.93	213.00	0.35	0.02	0.02	−0.02	0.06
	Shifting cultivation land area (ha)	2.44	0.12	0.77	213.00	0.44	0.01	0.01	−0.02	0.04
	Garden land area (ha)	0.79	0.37	0.53	213.00	0.60	0.00	0.01	−0.01	0.01
	Forestland area (ha)	16.82	0.00	3.86	213.00	0.00	1.51	0.34	0.83	2.19
	Pond land area (ha)	5.17	0.02	1.49	213.00	0.14	0.02	0.01	−0.01	0.04
	Residential land area (ha)	1.92	0.17	1.00	213.00	0.32	0.01	0.01	−0.01	0.03
	Other land area (ha)	2.11	0.15	0.73	213.00	0.47	0.01	0.02	−0.02	0.05
Loan	Taking out a loan from banks for production	0.95	0.33	1.64	213.00	0.10	0.12	0.07	−0.02	0.26
	Borrowing money from friends for production	0.82	0.37	0.44	213.00	0.66	0.03	0.07	−0.11	0.17
Labor	Number of family members	10.41	0.00	−4.08	213.00	0.00	−0.88	0.24	−1.36	−0.40
	Number of males	4.31	0.04	−2.59	213.00	0.01	−0.39	0.16	−0.71	−0.07
	Number of females	11.87	0.00	−3.21	213.00	0.00	−0.48	0.17	−0.80	−0.15
	Number of working people	1.02	0.31	−0.69	213.00	0.49	−0.11	0.15	−0.40	0.19
Social relationship	Holding position in a local organization	5.31	0.02	1.12	213.00	0.27	0.05	0.05	−0.04	0.14
Revenue	Total income ($)	1053.05	0.00	430.15	213.00	0.00	2071.30	170.71	1735.75	2407.30
	From rice ($)	50.42	12.94	40.61	213.00	0.36	19.63	21.42	−22.76	62.02
	From corn ($)	169.11	2.23	−44.17	213.00	0.33	−1.78	1.78	−5.80	1.78
	From potato ($)	20.53	22.31	−15.62	213.00	0.72	−0.45	1.34	−2.68	1.78
	From cassava ($)	33.91	16.96	19.19	213.00	0.67	0.89	1.78	−2.68	4.46
	From soybean ($)	182.05	1.78	44.62	213.00	0.32	3.12	3.12	−3.12	9.37
	From fruit ($)	331.98	0.45	64.70	213.00	0.15	56.22	38.37	−20.08	132.08
	From livestock ($)	595.24	0.00	158.40	213.00	0.00	389.99	90.58	211.50	568.47
	From seafood ($)	224.44	1.34	54.44	213.00	0.22	11.60	9.37	−7.14	30.34
	From forest ($)	1072.24	0.00	155.73	212.00	0.00	332.43	178.09	178.48	486.37
	From NTFPs ($)	92.81	6.69	32.13	213.00	0.47	1.78	2.68	−3.12	7.14
	From wages (labor) ($)	1111.06	0.00	298.96	213.00	0.00	1171.75	139.66	895.99	1445.72
	From doing business ($)	379.28	0.00	64.70	213.00	0.15	55.33	38.37	−20.08	131.19
	From official salary ($)	196.78	0.00	46.85	213.00	0.29	49.98	47.74	−43.73	143.68
	From pension ($)	10.71	27.66	−12.49	213.00	0.78	−12.94	45.51	−102.63	76.75

Moreover, revenue from plantation forests (Table 1) also made up a significant proportion, contributing to the overall income gap. Another factor was the number of members in a family. On average, poor households have 0.88 more people than non-poor ones. On the contrary, the ratio of workers generating income did not differ much between the two groups. According to Table 1, loans played a vital role in local people's economic development, with around 50% taking out some. However, non-poor households opted for a bank loan 12% higher than their poor counterparts.

## 2 Experimental Design, Materials and Methods

**Experimental design**: We employed the probability sample approach to make inferences beyond the sample in this study. Accordingly, we selected Chau Thai Commune as a study area for data collection for two reasons. First, Chau Thai is one of 206 poor communes in Nghe An [7]. Second, with the current forestland available, the commune has a high potential for afforestation, along with projects on climate change mitigation [11], [12]. After consulting local authorities about representative villages, we selected 4 villages: Dong Minh, Ban Hat, Thai Quang, and Dong Hin.

**Methods**: We conducted a survey in 2018 to obtain primary data on farmers’ livelihoods and perceptions of difficulties regarding the dimensions of livelihood, following the methods of [3], [13], [14], [15]. We adopted three steps in designing our study. First, we formed a focus group to help interviewers grasp the data collection procedure and enhance the questionnaire with the aid of the focus group members [9]. Second, we ran a pilot survey with a view to making proper adjustment to the questionnaire until the final version came out, with a total of 62 questions. The first part of the questionnaire, with 9 questions, investigates a households’ land use structure. The second part, 15 questions, looks into income structure. Next is people's perceptions towards setbacks in production forest planting, with 23 five-point likert scale questions [15], [16]. These questions were designed to obtain the different levels of plantation forest constraints. For example, ‘price of forest products are volatile’ is shown on a scale of five points where 1 and 5 refer to the lowest and highest level of agreement of respondents to the statement, respectively. The last part, with 15 questions, aims to collect the respondents’ personal information and socioeconomic background. Third, we conducted a survey using a random sampling technique and questionnaire-based face-to-face interviews [14]. We held a progress check to keep track and hold a meeting with survey team members at the end of each working day during the survey process. In total, 215 households living in 4 selected villages were interviewed. The data were then entered, converted to .xlsx format and coded for further analysis.

To capture the features of the respondents’ livelihood and their perceptions towards the constraints presented in Tables 1-2, we employed descriptive statistics to obtain results of mean, standard deviation, standard error, minimum and maximum values, and range. Furthermore, we also ran a One-sample T-test to obtain a confidence interval for mean (95%). Regarding Table 3, we compare means via independent sample T-test, including Levene's test for equality of variances and a T-test for equality of means, with confidence interval of the difference being 95%. The rationale lies with the fact that the impoverished is the research target and this way of categorization facilitates comparison between these two groups. We could explore the differences in land and income structure, demographic features, and perceptions.

As for this research, there is still room for improvement, and acknowledgement of the limitations allows the research quality to improve [17]. First, the sample size is only 215 households, which means that its confidence level is just above 85%, and the error level is 5% (Table 4). However, for normally distributed data, a number of observations of 50 or more are needed to have reasonably short confidence bounds on the variance estimate [18]. Thus, our size could be deemed large enough to undergo data processing and analysis. Second, the study region is in a poor and mountainous commune where the literacy level of the interviewees is limited, causing difficulties for interviewers to some degree. In addition, rural household livelihood associated with forest development is influenced by many socioeconomic factors, especially environmental and cultural values [19]. Yet, its absence is a limitation of research and should be considered in future studies.

**Table 4:** Minimum sample size at various confidence and error levels.

Confidence level		80%	85%	90%	95%
Error level	1.0%	4096	5148	6766	9604
	2.0%	1024	1296	1692	2401
	3.0%	456	576	752	1068
	4.0%	256	324	423	601
	5.0%	164	**208**	271	385
	7.5%	73	93	121	171

## Ethics Statement

The authors declare that this study is designed for the purpose of research and goodwill. Interviews are conducted on the basis of willingness and mutual consent, and participants’ personal information remain confidential.

## CRediT Author Statement

**Quan-Hoang Vuong:** Conceptualization, Methodology, Formal analysis, Supervision and validation, Writing - Original draft, Writing - Review & editing; **Phu Pham:** Conceptualization, Methodology, Formal analysis, Writing - Original draft, Writing - Review & editing; **My-Hien Nguyen:** Formal analysis, Writing - Original draft, Writing - Review & editing; **Cong-Thang Ngo:** Formal analysis, Visualization, Writing - Original draft, Writing - Review & editing; **Phuong-Mai Tran:** Formal analysis, Writing - Original draft, Writing - Review & editing; **Quy Van Khuc:** Conceptualization, Methodology, Data curation, Project Administration, Formal analysis, Supervision and validation, Writing - Original draft, Writing - Review & editing. All authors have read and agreed to the version of the manuscript.

## Declaration of Competing Interest

The authors declare that they have no known competing financial interests or personal relationships that could have influenced the work reported in this paper.

## Appendix Supplementary materials

Image, application 1

Image, application 2
