# Supplementary material for: Farmers’ livelihood strategies and perceived constraints from poor and non-poor households: A dataset from a field survey in Nghe An, Vietnam
Source: Data Brief. 2021 Mar 31;36:106991. doi: 10.1016/j.dib.2021.106991 (PMC8079539; doi:10.1016/j.dib.2021.106991)
Supplement: Supplementary file 2 [file mmc2.docx]


**Farmers' livelihood strategies and perceived constraints from the poor and non-poor households: A dataset from field survey in Nghe An, Vietnam**

| 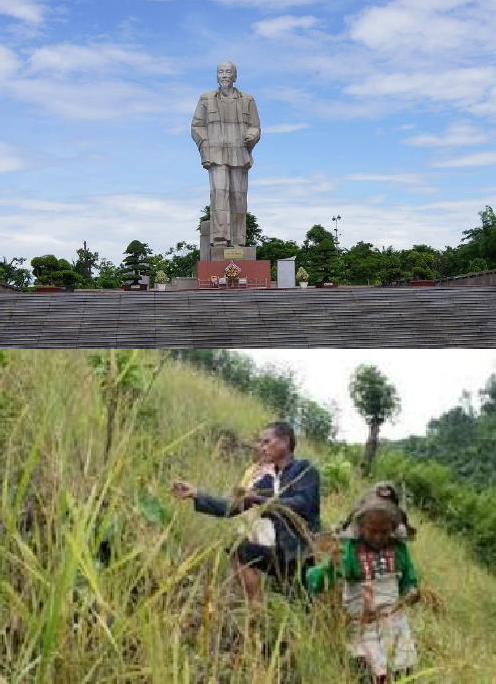 | 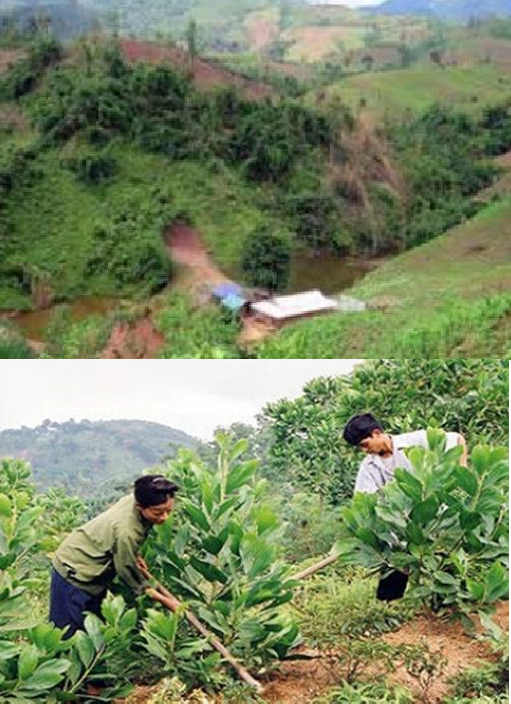 |
| --- | --- |

| **Structure of the questionnaire** | | **Number of question** | **Pages** |
| --- | --- | --- | --- |
| Part A | Household land use structure | 9 | 2 |
| Part B | Household income structure | 15 | 3 |
| Part C | Perceived factors hindering production forest planting | 23 | 4 |
| Part D | Household information | 15 | 7 |
| Total pages | |  | 8 |

- Investigation team: Vietnamese Academy of Forest Sciences, Vietnam National University of Forestry, North Central Hydrometeorological Center
- We appreciate your time to help complete this questionnaire.
- The information collected is only used to serve scientific research purposes. We will not, in any circumstances, share your information with other individuals or organizations without your consensus.

Hanoi, 2018

**PART A: Land use structure of your family (please circle unit of measurement: ha or m^2^/ (3) will undergo data processing later)**

|  | **Land** | **Area corresponding to number of plots (ha or m2)** | | | | | **Total area (ha)** |
| --- | --- | --- | --- | --- | --- | --- | --- |
|  | **(1)** | **(2.1)** | **(2.2)** | **(2.3)** | **(2.4)** | **(2.5)** | **(3)** |
| **A1** | **Wet rice land** |  |  |  |  |  |  |
| **A2** | **Paddy rice land** |  |  |  |  |  |  |
| **A3** | **Shifting cultivation land** |  |  |  |  |  |  |
| **A4** | **Garden land** |  |  |  |  |  |  |
| **A5** | **Forest land** |  |  |  |  |  |  |
| **A6** | **Pond land** |  |  |  |  |  |  |
| **A7** | **Residential land** |  |  |  |  |  |  |
| **A8** | **Other** |  |  |  |  |  |  |
| **A9** | **Total** |  |  |  |  |  |  |

**PART B: Detailed information on and origin of household INCOME (2017)**

|  | **Category** | **Income** | | | | | |
| --- | --- | --- | --- | --- | --- | --- | --- |
|  |  | ***Total sum*** | **Unit** | **Quantity** | **Unit** | **Total**  ***(million VND)*** | **Note** |
| **B1** | **Rice** |  |  |  |  |  |  |
| **B2** | **Corn** |  |  |  |  |  |  |
| **B3** | **Potato** |  |  |  |  |  |  |
| **B4** | **Cassava** |  |  |  |  |  |  |
| **B5** | **Soybean** |  |  |  |  |  |  |
| **B6** | **Fruit** |  |  |  |  |  |  |
| **B7** | **Livestock** |  |  |  |  |  |  |
| **B8** | **Seafood** |  |  |  |  |  |  |
| **B9** | **Planted forests** |  |  |  |  |  |  |
| **B10** | **Non-timber forest products** |  |  |  |  |  |  |
| **B11** | **Wage earning** |  |  |  |  |  |  |
| **B12** | **Doing business** |  |  |  |  |  |  |
| **B13** | **Official salary** |  |  |  |  |  |  |
| **B14** | **Pension** |  |  |  |  |  |  |
| **B15** | **Total** |  |  |  |  |  |  |

**PART C. Factors hindering production forest planting among households**

***How to fill: Please highlight one of the options ①, ②, ③, ④ or ⑤ to the extent that you agree with the statements according to the following levels:***

***① - Totally disagree ② - Disagree ③ - Neutral ④ - Agree ⑤ - Totally agree***

| **Interviewees:**  Households who (1) have forest land and have been planting forests, (2) or have shifting cultivation land that can be converted to plant forests |
| --- |

**Section 1: Group of Land Factors (5 questions)**

C1. The area of forest land is small for your family.

|  | 1 | 2 | 3 | 4 | 5 |  |
| --- | --- | --- | --- | --- | --- | --- |
| Totally disagree |  |  |  |  |  | Totally agree |

C2. Forest land is poor or infertile.

|  | 1 | 2 | 3 | 4 | 5 |  |
| --- | --- | --- | --- | --- | --- | --- |
| Totally disagree |  |  |  |  |  | Totally agree |

C3. The location of the forest land is far away from your home.

|  | 1 | 2 | 3 | 4 | 5 |  |
| --- | --- | --- | --- | --- | --- | --- |
| Totally disagree |  |  |  |  |  | Totally agree |

C4. Your forest land is steep, thereby adversely affecting production.

|  | 1 | 2 | 3 | 4 | 5 |  |
| --- | --- | --- | --- | --- | --- | --- |
| Totally disagree |  |  |  |  |  | Totally agree |

C5. Your family is not reassured to cultivate on this forest land (as all or part of forest land/ land for afforestation does not have clear boundaries, or there is no Land Ownership Certification, etc.)

|  | 1 | 2 | 3 | 4 | 5 |  |
| --- | --- | --- | --- | --- | --- | --- |
| Totally disagree |  |  |  |  |  | Totally agree |

**Section 2: Group of Capital Factors (5 questions)**

Did you take out a loan for afforestation? □ Yes □ No

**If Yes,** continue with question 6

**If No,** continue with question 10

C6. Taking out the loan from banks, friends and others is difficult

|  | 1 | 2 | 3 | 4 | 5 |  |
| --- | --- | --- | --- | --- | --- | --- |
| Totally disagree |  |  |  |  |  | Totally agree |

C7. The amount of capital that is allowed to borrow or borrowed is little, or not enough to meet the family's needs.

|  | 1 | 2 | 3 | 4 | 5 |  |
| --- | --- | --- | --- | --- | --- | --- |
| Totally disagree |  |  |  |  |  | Totally agree |

C8. In your opinion, the loan period is short.

|  | 1 | 2 | 3 | 4 | 5 |  |
| --- | --- | --- | --- | --- | --- | --- |
| Totally disagree |  |  |  |  |  | Totally agree |

C9. In your opinion, the loan interest rate is high.

|  | 1 | 2 | 3 | 4 | 5 |  |
| --- | --- | --- | --- | --- | --- | --- |
| Totally disagree |  |  |  |  |  | Totally agree |

C10.Your family has no or little equity capital.

|  | 1 | 2 | 3 | 4 | 5 |  |
| --- | --- | --- | --- | --- | --- | --- |
| Totally disagree |  |  |  |  |  | Totally agree |

**Section 3: Group of Labor factors (5 questions)**

C11. The number of workers in the family is small.

|  | 1 | 2 | 3 | 4 | 5 |  |
| --- | --- | --- | --- | --- | --- | --- |
| Totally disagree |  |  |  |  |  | Totally agree |

C12. The family's labor suffers from poor health.

|  | 1 | 2 | 3 | 4 | 5 |  |
| --- | --- | --- | --- | --- | --- | --- |
| Totally disagree |  |  |  |  |  | Totally agree |

C13. The Head of Household has a poor educational background.

|  | 1 | 2 | 3 | 4 | 5 |  |
| --- | --- | --- | --- | --- | --- | --- |
| Totally disagree |  |  |  |  |  | Totally agree |

C14. The Head of Household has little knowledge and experience in afforestation

|  | 1 | 2 | 3 | 4 | 5 |  |
| --- | --- | --- | --- | --- | --- | --- |
| Totally disagree |  |  |  |  |  | Totally agree |

C15. The Head of Household has a fragile relationship with people around (business partners), or does not hold a position of authority.

|  | 1 | 2 | 3 | 4 | 5 |  |
| --- | --- | --- | --- | --- | --- | --- |
| Totally disagree | 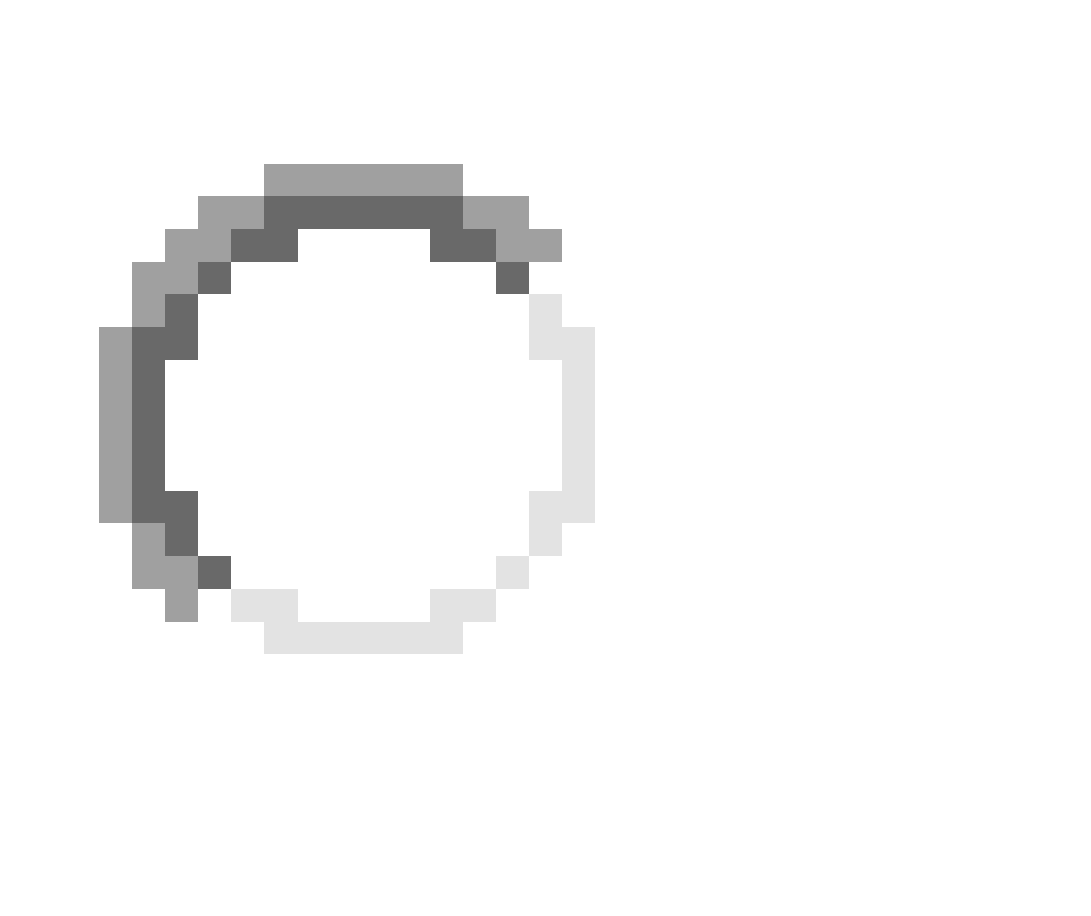 | 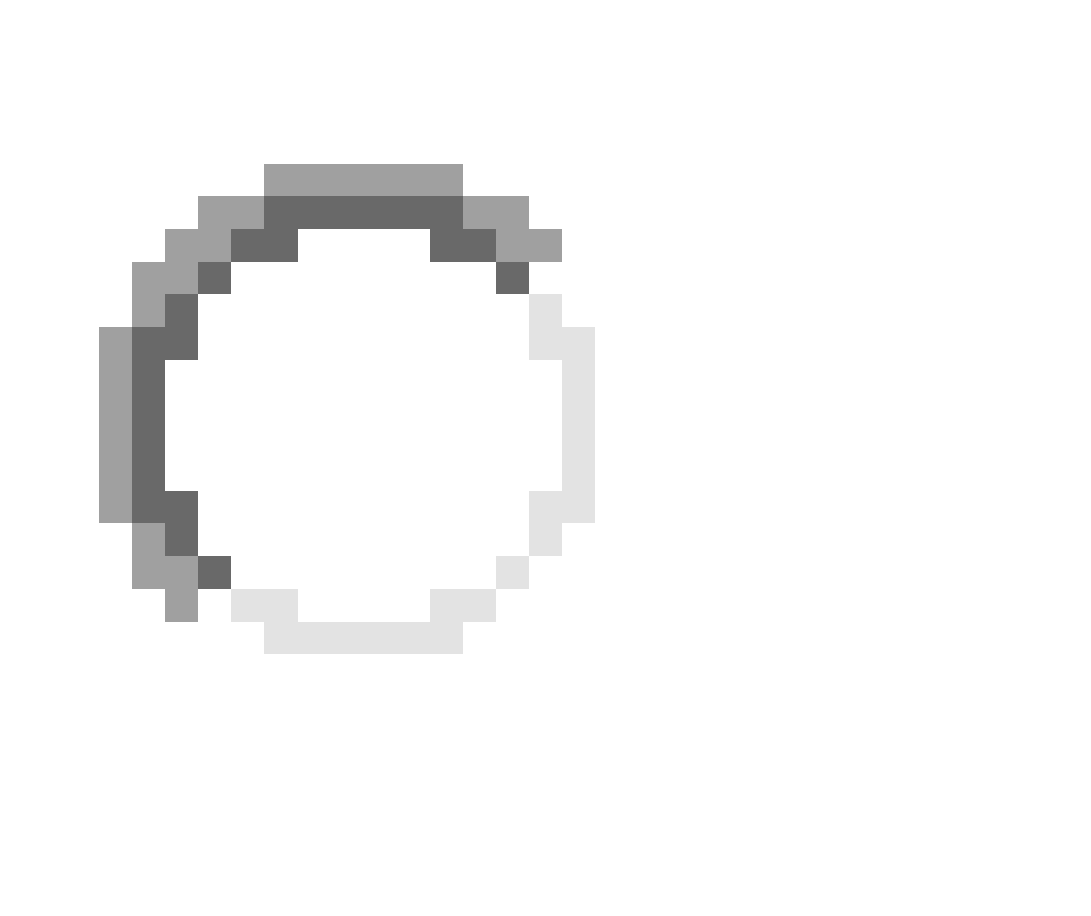 | 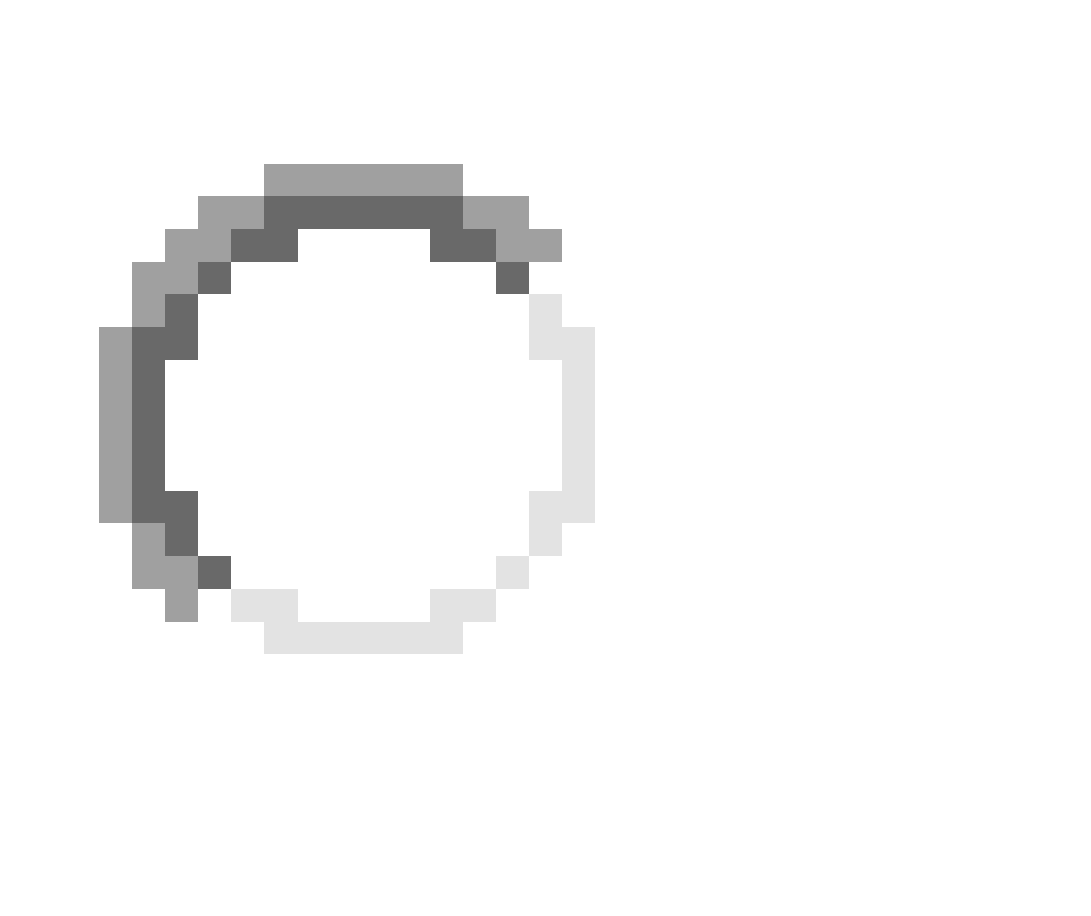 | 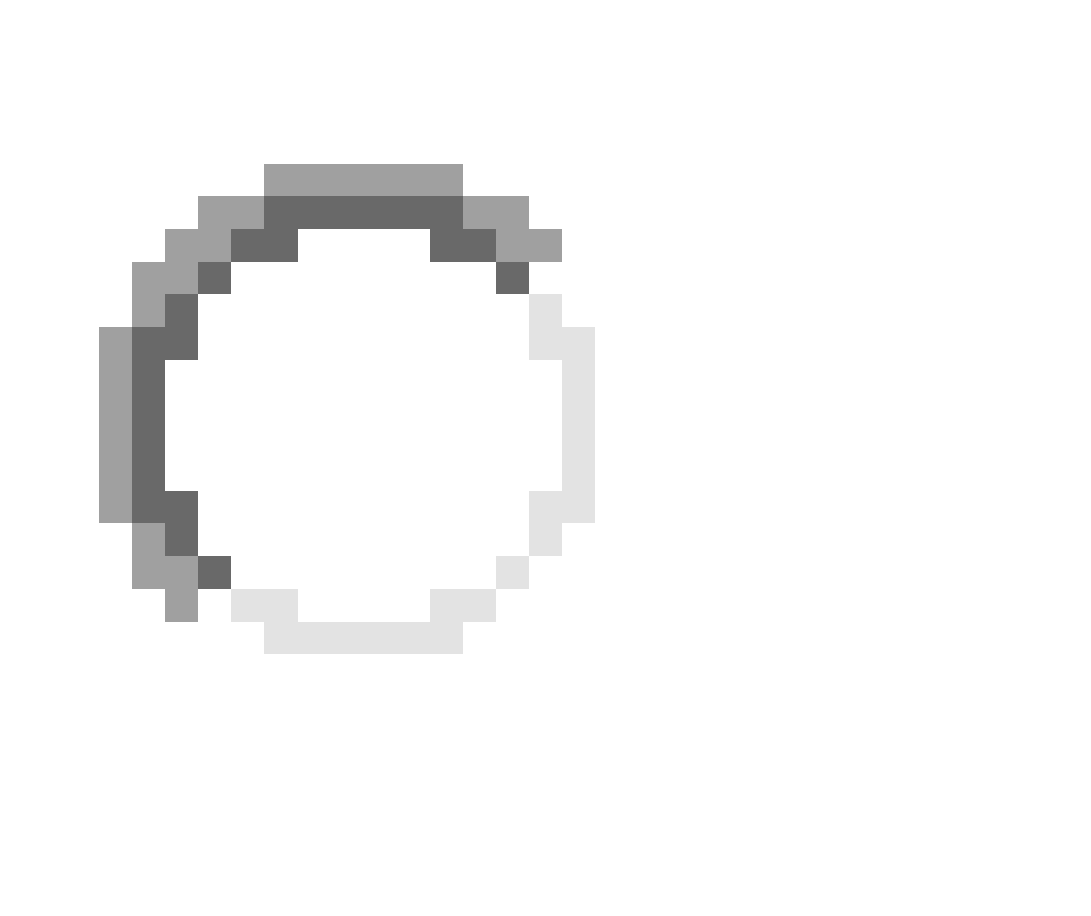 | 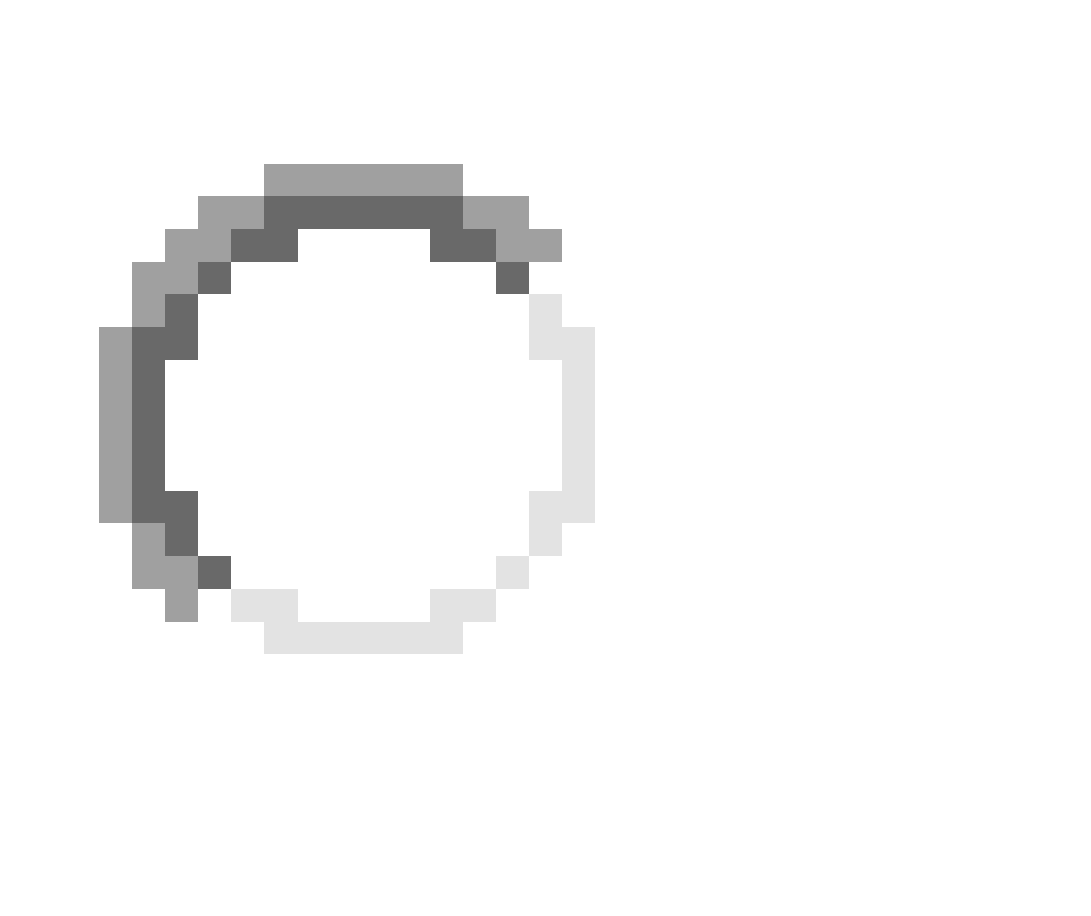 | Totally agree |

**Section 4: Group of Forest products market factors (5 questions)**

C16. Information on selling prices of products from planted forests (forest timber) is unclear.

|  | 1 | 2 | 3 | 4 | 5 |  |
| --- | --- | --- | --- | --- | --- | --- |
| Totally disagree |  |  |  |  |  | Totally agree |

C17. In your opinion, selling prices of forest timber are volatile.

|  | 1 | 2 | 3 | 4 | 5 |  |
| --- | --- | --- | --- | --- | --- | --- |
| Totally disagree |  |  |  |  |  | Totally agree |

C18. In your opinion, selling prices of timber products are often low in your place.

|  | 1 | 2 | 3 | 4 | 5 |  |
| --- | --- | --- | --- | --- | --- | --- |
| Totally disagree |  |  |  |  |  | Totally agree |

C19. Wholesalers drive a hard bargain.

|  | 1 | 2 | 3 | 4 | 5 |  |
| --- | --- | --- | --- | --- | --- | --- |
| Totally disagree |  |  |  |  |  | Totally agree |

C20. The market of manufacturers is weak (unstable, limited purchasing power,etc.)

|  | 1 | 2 | 3 | 4 | 5 |  |
| --- | --- | --- | --- | --- | --- | --- |
| Totally disagree | 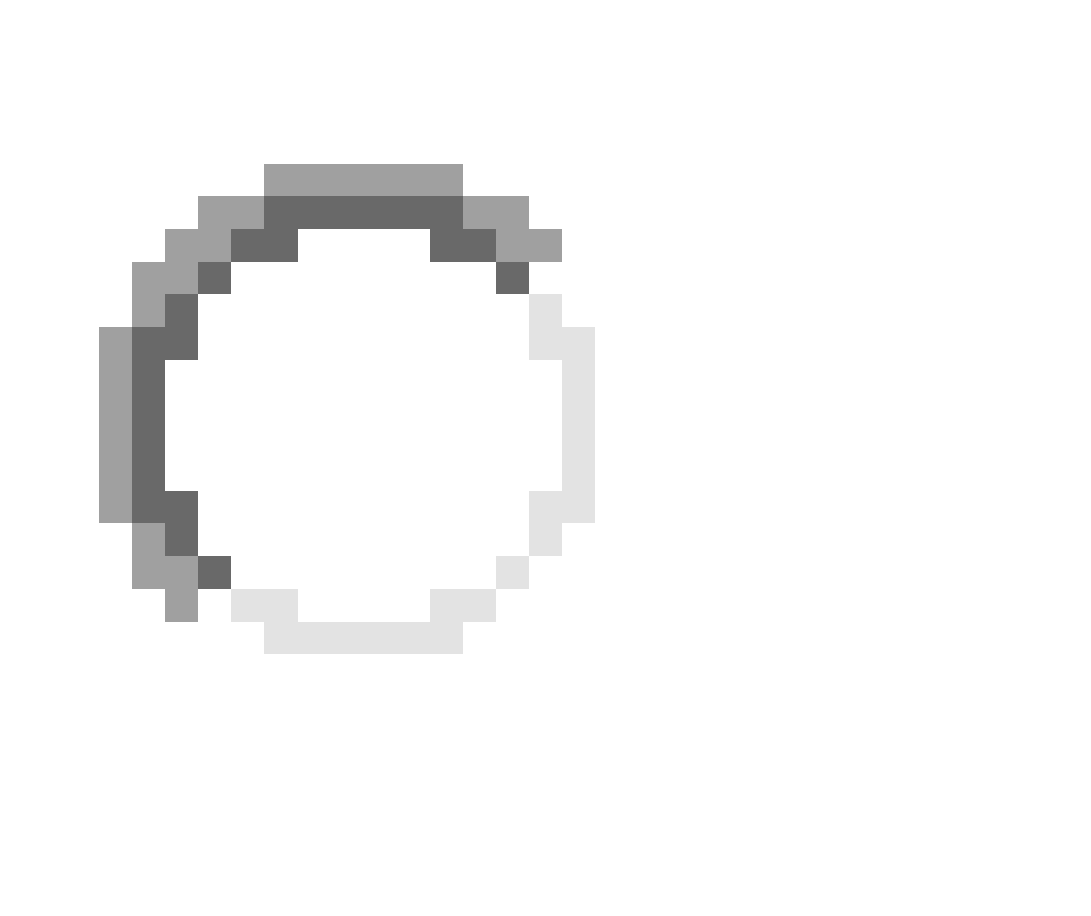 | 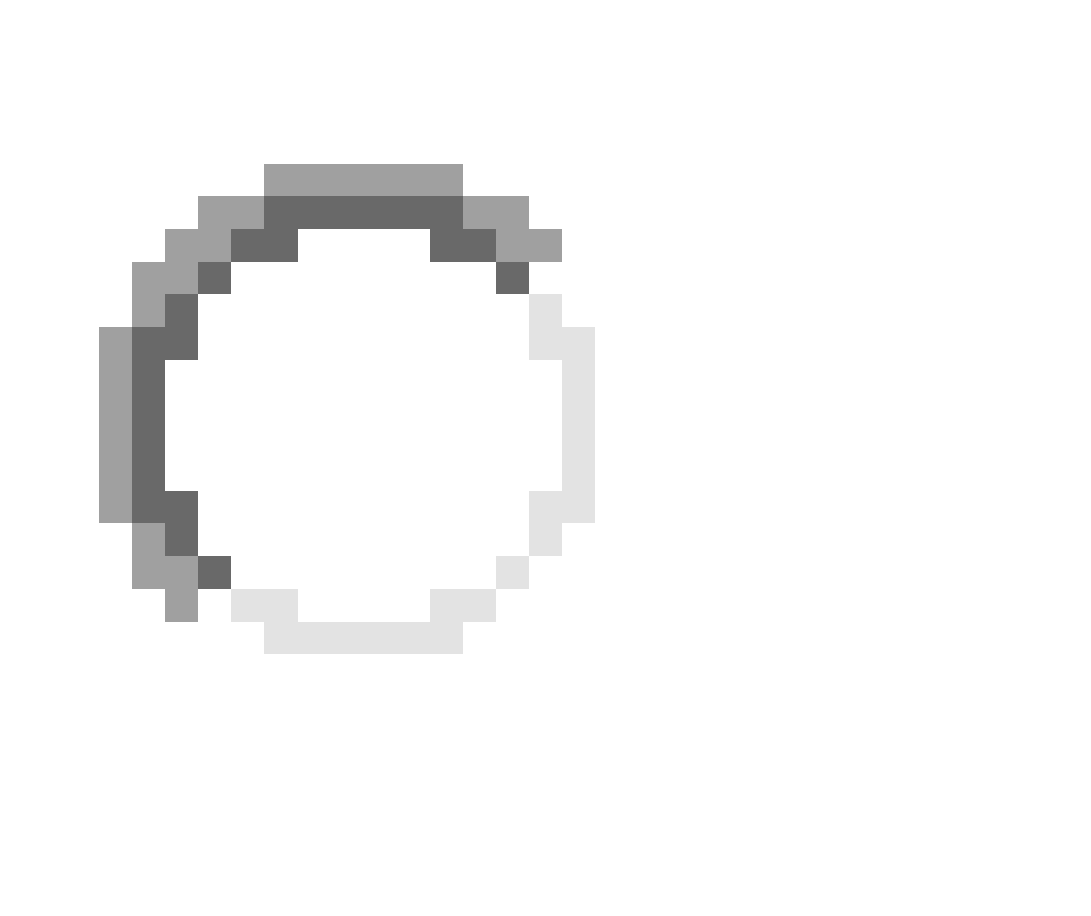 | 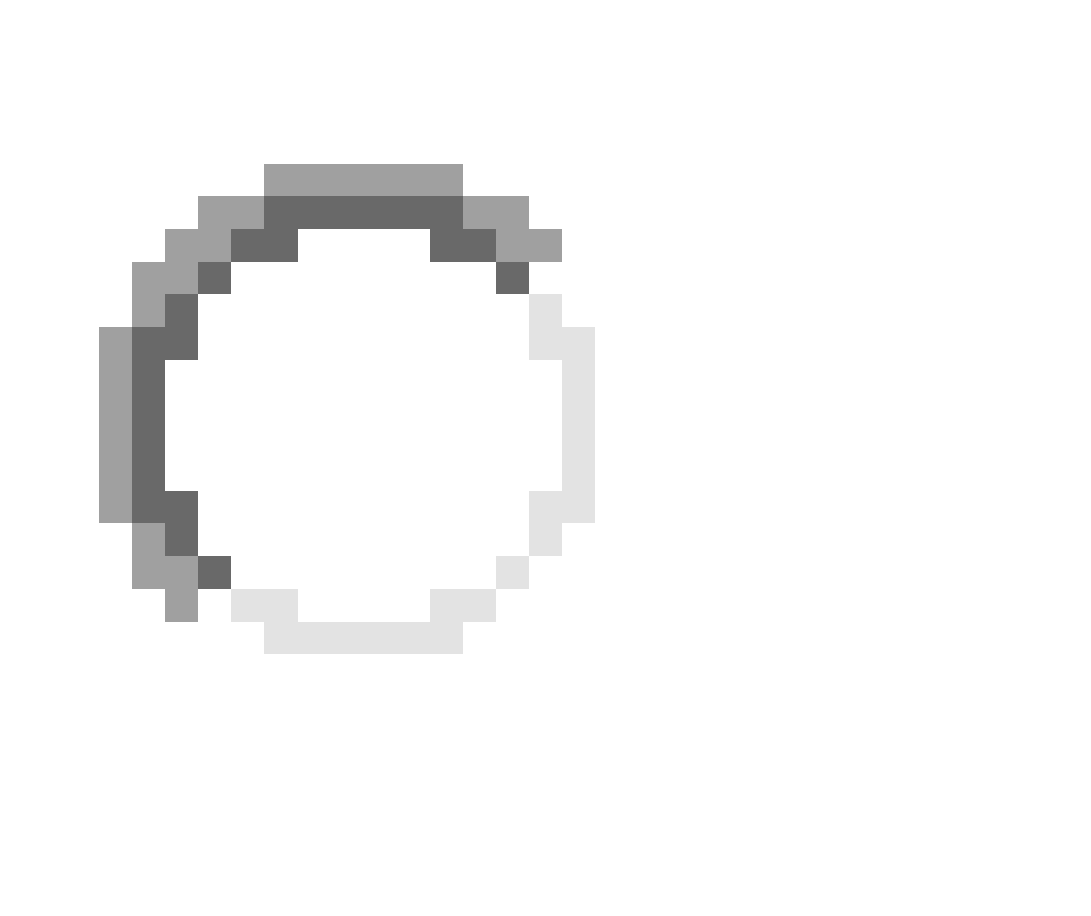 | 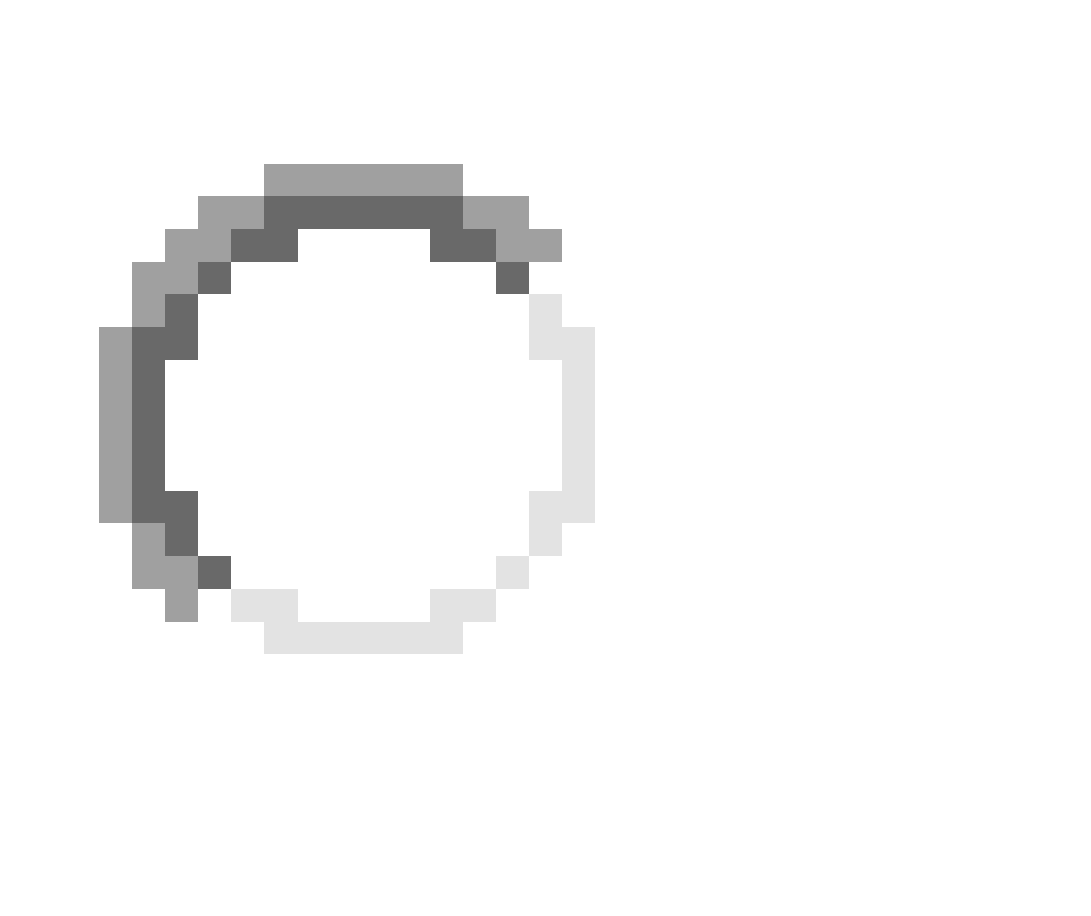 | 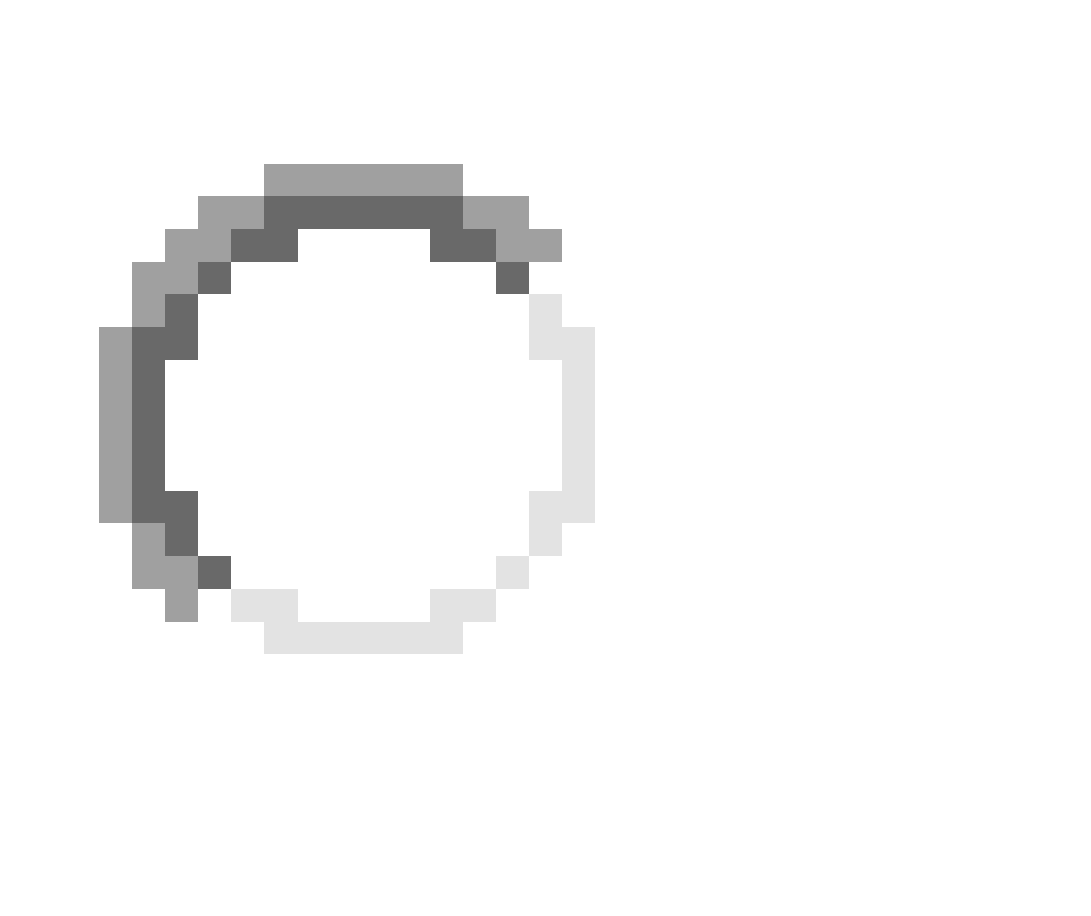 | Totally agree |

**Section 5: General evaluation (3 questions)**

C21. Your family has difficulty with forest land.

|  | 1 | 2 | 3 | 4 | 5 |  |
| --- | --- | --- | --- | --- | --- | --- |
| Totally disagree |  |  |  |  |  | Totally agree |

C22. Your family has difficulty with human resources for afforestation

|  | 1 | 2 | 3 | 4 | 5 |  |
| --- | --- | --- | --- | --- | --- | --- |
| Totally disagree |  |  |  |  |  | Totally agree |

C23. Your family feels that you have or will have difficulty selling products from the forest.

|  | 1 | 2 | 3 | 4 | 5 |  |
| --- | --- | --- | --- | --- | --- | --- |
| Totally disagree |  |  |  |  |  | Totally agree |

**PART D: Household Information (If not asked at the beginning, ask at the end) (12 questions)**

Please fill in the personal information of the interviewee

| Full name of head of household _____________________  Home phone number ___________  Mobile phone number ____________  Email _____________ | Address of your current home________ | |
| --- | --- | --- |
| D1. Do you belong to ethnic minority group? | 🗆 Yes | 🗆 No |
| D2. Gender | 🗆 Male | 🗆 Female |
| D3. Age _________ |  |  |
| D4. Total years of schooling ________________ |  |  |

D5. Number of people in your family: ______________________________

D6. How many men: ____________ D7. How many women: ________

D8. How many people in your family are **the main labor** (with current income, excluding retirees) ________ (unit: person)

D9. How many years have you been living in your current place? ____________year

D10. How many years has the head of household been doing farming (current job)?_______;

D11. Have you participated in afforestation technique training (over the past 5 years)?

🗆Yes 🗆No

D12. Have you participated in agricultural technique training (over the past 5 years)?

🗆Yes 🗆No

D13. Have you taken out a bank loan to boost production?

🗆Yes 🗆No

D14. Have you borrowed money from friends or other acquaintances to boost production?

🗆Yes 🗆No

D15. Do you hold any position in any local organization (village, commune, etc.)?

🗆Yes 🗆No

If Yes, the position title is:_____

**_________ End of the questionnaire, thank you very much for your help_______**
